# Incidence of Gallstones in Patients with Obesity After Bariatric Surgery in Northern Saudi Arabia: A Cross-Sectional Study

**DOI:** 10.3390/clinpract15070115

**Published:** 2025-06-23

**Authors:** Abdulrahman Omar A. Alali, Abdualaziz Fayez Alhumidi Alanazi, Mohammed Abdulaziz M. Albarghash, Rakan Nasser Abdullah Alruweli, Mohammed Bader H. Alanazi, Ibrahim Farhan B. Alanazi, Turkey Saleh H. Alrowaily, Rakan Khalid Marzouq Alanazi, Baraah AbuAlsel, Fadih Nada M. Alenezi, Rashad Qasem Ali Othman, Manal S. Fawzy

**Affiliations:** 1 Faculty of Medicine, Northern Border University, Arar 91431, Saudi Arabia; dr.a.o.alali@gmail.com (A.O.A.A.); azoozov501@gmail.com (A.F.A.A.); abdulazizm0400@gmail.com (M.A.M.A.); rakanraweli@gmail.com (R.N.A.A.); m8baderm8@gmail.com (M.B.H.A.); alnzy1333@gmail.com (I.F.B.A.); yled97@gmail.com (T.S.H.A.); rakanbbgg1212@gmail.com (R.K.M.A.); 2 Department of Pathology, Faculty of Medicine, Northern Border University, Arar 91431, Saudi Arabia; baboalseel@nbu.edu.sa; 3 Bariatric Surgery Center, Prince Abdulaziz Bin Musaad Hospital, Arar 73212, Saudi Arabia; fadih1995@gmail.com; 4 Center for Health Research, Northern Border University, Arar 73213, Saudi Arabia

**Keywords:** bariatric surgery, cholelithiasis, gallstones, incidence, obesity, risk factors, Northern Border Region

## Abstract

Background/Objectives: Gallstone formation (cholelithiasis) is a common and important consequence following bariatric surgery, though regional data from the Northern Border Region are limited. This study aimed to investigate the incidence and risk factors of gallstones in this population, with the goal of optimizing postoperative treatment and reducing morbidity. Methods: We conducted a cross-sectional study using a non-probability convenience sampling technique to recruit 509 participants with varying degrees of obesity. Four hundred and ten study participants underwent bariatric surgery, of whom 73 were excluded for preoperative cholelithiasis and/or cholecystectomy. Data were collected through a self-administered, pre-validated questionnaire distributed via various social media platforms. These data included demographics, type/timing of surgery, pre/postoperative BMI, medical history, use of gallstone prophylaxis, and gallstone outcomes. Logistic regression analysis was used to identify independent predictors of gallstone formation. Results: Postoperative cholelithiasis developed in 60.8% of patients, most commonly within the first postoperative year, with risk peaking between 7 and 12 months after surgery. Rapid and substantial postoperative weight loss, as reflected in a lower current BMI and a transition to normal or overweight status within one year, was significantly associated with an increased incidence of gallstones. Female sex (OR: 2.62, 95% CI: 1.38–4.98, *p* = 0.003) and non-use of gallstone prevention medication (OR: 4.12, 95% CI: 1.34–12.64, *p* = 0.013) were independent predictors of gallstone formation. A longer time since surgery (OR: 0.76, 95% CI: 0.63–0.91, *p* = 0.004) and a lower current BMI (OR: 0.48, 95% CI: 0.28–0.83, *p* = 0.008) were associated with a reduced risk. Smoking status and comorbidities were not significantly related to the risk of gallstones. Conclusions: Gallstone formation after bariatric surgery in this population is influenced by female sex, rapid postoperative weight loss, and lack of prophylactic medication, while the type of surgical procedure does not significantly affect risk. Focused monitoring and preventive strategies, particularly in high-risk groups, are recommended to reduce gallstone-related complications following bariatric surgery.

## 1. Introduction

Obesity is a global epidemic and a major public health concern, with prevalence rates rising sharply in many countries, including Saudi Arabia [1]. Obese individuals are at increased risk for a spectrum of comorbidities, such as cardiovascular disease, hyperlipidemia, type 2 diabetes, and gallstone disease (cholelithiasis) [2]. Among these, gallstone formation is particularly noteworthy due to its association with rapid weight loss, which commonly follows very low-calorie diets or bariatric surgery interventions [3].

Gallbladder disease, especially cholecystitis secondary to gallstones, is one of the most frequent surgical problems encountered in general surgery [4]. Cholelithiasis refers to the presence of abnormal concretions (gallstones) within the gallbladder, with obesity recognized as a significant risk factor for their development [5]. The pathogenesis of gallstones in obese individuals is multifactorial, involving cholesterol supersaturation of bile, impaired gallbladder motility, and metabolic alterations [6]. Rapid weight loss, as seen after bariatric procedures, further exacerbates these risks by enhancing cholesterol mobilization and altering biliary composition [7,8].

Bariatric surgery, including sleeve gastrectomy (SG) and Roux-en-Y Gastric Bypass (RYGB), is the most effective long-term intervention for obesity and its related comorbidities [9]. However, these procedures are associated with a substantial risk of gallstone formation, particularly within the first two years postoperatively [3,7]. The incidence of gallstones after bariatric surgery varies widely, with rates reported between 10.4% and 52.8% in the first postoperative year, depending on the surgical technique and patient population [3,10]. Notably, RYGB is generally associated with a higher risk of cholelithiasis compared to SG, likely due to greater and more rapid weight loss, as well as potential injury to the hepatic branch of the vagus nerve during surgery, which can impair gallbladder motility and promote bile stasis [10].

Most gallstones formed postoperatively are cholesterol stones, resulting from bile supersaturation, reduced bile acid secretion, increased mucus production, and decreased gallbladder emptying. Symptomatic gallstones can lead to significant morbidity, including biliary colic, cholecystitis, cholangitis, pancreatitis, and, in rare cases, gallbladder carcinoma. While some patients remain asymptomatic, the risk of progression to severe complications necessitates vigilant postoperative monitoring and management [11,12].

Preventive strategies for gallstone formation after bariatric surgery include pharmacological interventions such as ursodeoxycholic acid (UDCA), which has demonstrated efficacy in reducing gallstone incidence and the need for subsequent cholecystectomy [3]. Prophylactic cholecystectomy during bariatric surgery is another consideration, particularly for patients with pre-existing gallstones, though its routine use remains debated due to operative risks and resource implications [13]. Dietary factors, including increased fiber, healthy fats, and certain supplements, may also play a role in reducing gallstone risk, but evidence for their effectiveness in the bariatric population is limited [14,15,16].

Despite the global and national burden of obesity and gallstone disease, there is a notable lack of region-specific data, particularly in Northern Saudi Arabia [17]. Existing studies have predominantly focused on Western or central regions, with little attention to the unique demographic, dietary, and healthcare factors influencing gallstone incidence in the Northern Border Region. This gap in the literature underscores the need for localized research to inform clinical practice and optimize postoperative care for bariatric patients in this area.

Therefore, the present cross-sectional study aims to determine the incidence of gallstone formation in obese patients following bariatric surgery in the Northern Border Region of Saudi Arabia. By elucidating the frequency and risk factors associated with post-bariatric cholelithiasis in this specific population, our findings will contribute valuable insights to the development of tailored postoperative management strategies, ultimately improving patient outcomes and reducing the burden of gallstone-related complications in this high-risk group.

## 2. Materials and Methods

### 2.1. Study Design and Setting

This cross-sectional study was conducted in the Northern Border Region of Saudi Arabia to determine the incidence of gallstone formation in obese patients following bariatric surgery. The study adhered to the “STrengthening the Reporting of OBservational studies in Epidemiology” (STROBE) guidelines for observational research [18]. Data were collected over 3 months (June–September 2024) via several online platforms.

### 2.2. Participant Selection

The criteria for participant selection from the overall respondents were adults aged 18–60 years with a history of bariatric surgery (including sleeve gastrectomy, Roux-en-Y gastric bypass, adjustable gastric banding, or other bariatric procedures) prior to enrollment, who resided in the specified study region. Participants were excluded if they had a preoperative diagnosis of cholelithiasis, a history of cholecystectomy prior to undergoing bariatric surgery, incomplete response records, or if they refused to participate. Informed written consent was obtained from all participants prior to their participation.

### 2.3. Sampling Strategy

A convenience sample of at least 323 participants was calculated using Raosoft’s sample size calculator, available at http://www.raosoft.com/samplesize.html (accessed on 5 March 2024), with a 95% confidence interval, a 5% margin of error, a population size of the Northern area of 390,656, and a response distribution of 70%. The sample reflected demographic diversity across gender, nationality, and socioeconomic status within the region.

### 2.4. Data Collection

Data were collected using a structured, self-administered questionnaire in Arabic, adapted from previously validated instruments used in studies of gallstone incidence following bariatric surgery in Saudi Arabia to ensure content validity and relevance to the study objectives [19,20]. Specialists in bariatric surgery and epidemiology conducted the expert review. The current instrument enabled the collection of detailed demographic, clinical, surgical, and risk factor data necessary to assess the incidence and determinants of gallstone formation after bariatric surgery in the target population.

The questionnaire was distributed electronically via several social media platforms using “Google Forms.” It included the following main components: (1) Informed consent and study information: participants were first provided with detailed information about the study’s purpose, voluntary nature, confidentiality measures, and potential risks and benefits. Informed digital consent was obtained prior to participation. (2) Demographic data: participants reported their sex, age group (18–25, 26–35, 36–45, 46–55, 56–60 years), nationality (Saudi or non-Saudi), place of residence (Northern Region or other), educational level (from uneducated to postgraduate), marital status (single, married, divorced, widowed), occupation (student, employee, unemployed, retired, or other), and height (in centimeters). (3) Bariatric surgery and weight history: information was collected regarding the history of weight loss surgery (yes/no), date of surgery, type of bariatric procedure (gastric sleeve, Roux-en-Y gastric bypass, adjustable gastric banding, or other), and weight measurements at multiple time points: before surgery, at 3 months, 6 months, and 1-year post-surgery, as well as current weight (all in kilograms). (4) Gallstone history and diagnosis: participants were asked about any previous diagnosis of gallstones or cholecystectomy prior to bariatric surgery, whether a preoperative abdominal ultrasound was performed, and if they had been diagnosed with gallstones following surgery. For those diagnosed postoperatively, additional questions addressed the diagnostic modality (ultrasound/sonar) and the time interval from surgery to gallstone development (categorized as 1–3 months, 4–6 months, 7–9 months, 9–12 months, 1.5 years, 2 years, or more than 2 years). (5) Medical and lifestyle risk factors: the questionnaire included items on preoperative comorbidities (hypertension, diabetes, hypercholesterolemia, anemia, thyroid disease, asthma, chronic heart/liver/kidney disease, or other), smoking status (current, former, or never), and use of medications for gallstone prevention or dissolution during the first six months after surgery.

### 2.5. Variables and Measurements

The primary outcome of this study was the incidence of gallstones, confirmed through imaging techniques. The independent variables included the type of surgical procedure, preoperative body mass index (BMI) for calculating the percentage of total weight loss, and the duration of postoperative follow-up. The confounding control was adjusted for age, sex, and comorbidities.

### 2.6. Statistical Analysis

Data were analyzed using the “Statistical Package for Social Sciences (SPSS)” version 27 (IBM SPSS Statistics, Armonk, NY, USA). The distribution of the data was evaluated to determine the most suitable statistical analysis. Analytical approaches included (a) descriptive statistics that were reported as frequencies (%) and/or mean ± SD, (b) bivariate analysis by the chi-square test or Fisher’s exact test when appropriate for categorical associations or *T*-test for continuous data, and (c) logistic regression analysis to determine the predictors of gallstone formation after bariatric surgery. The significance threshold was set at *p* ≤ 0.05 (two-tailed).

## 3. Results

### 3.1. Participant Characteristics

**Figure 1:**
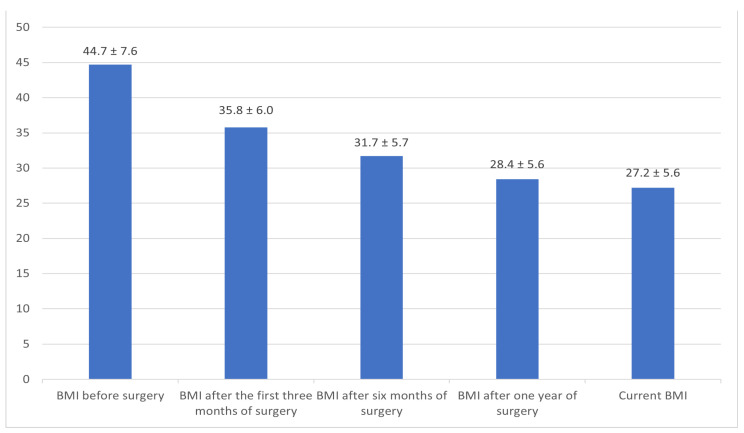
Pre-/post-obesity surgery body mass index (BMI) means ± SD at different intervals.

Of the 509 individuals recruited for this study, 410 participants (80.6%) reported having undergone bariatric surgery. From the latter group, 73 were excluded as they were diagnosed with gallstones or had undergone cholecystectomy before obesity surgery. The baseline demographic and clinical characteristics of all enrolled participants are summarized in Table 1. In terms of smoking status, 65.9% of participants who had bariatric surgery identified as non-smokers, 28.5% as current smokers, and 5.6% as former smokers. They had a mean preoperative BMI of 44.7 ± 7.6 kg/m^2^, which decreased significantly to 27.2 ± 5.6 kg/m^2^ following surgery (Figure 1 and Supplementary Table S1).

Regarding surgical procedures, sleeve gastrectomy (SG) was the predominant intervention, performed in 88.4% of cases, while Roux-en-Y gastric bypass (RYGB) accounted for 4.5% of surgeries (Table 2). The ‘Others’ surgical category included less commonly performed surgical procedures (e.g., fundoplication, mini-gastric bypass, vertical banded gastroplasty, and gastric plication), each representing fewer than five cases. These were grouped to avoid statistical overinterpretation of small subgroups.

### 3.2. Pre- and Postoperative Gallbladder Conditions

Table 3 depicts the frequency of gallbladder-related conditions before and after bariatric surgery. Prior to surgery, 192 participants (57.0%) had a preoperative abdominal ultrasound. After bariatric surgery, 205 patients (60.8%) were diagnosed with gallstones, with the majority (82.4%, n = 169) receiving their diagnosis by abdominal ultrasound.

### 3.3. Timing of Gallstone Development After Bariatric Surgery

Among participants who developed gallstones postoperatively (n = 205), the majority experienced gallstone formation within the first year after surgery. Specifically, 8.8% reported gallstone development within 1–3 months, 20.5% between 4 and 6 months, and a notable 45.4% between 7 and 9 months postoperatively. The distribution of gallstone onset was statistically significant (*p* < 0.001), indicating a clear trend toward early postoperative formation (Table 4).

### 3.4. Preoperative Medical Conditions

Regarding comorbidities prior to bariatric surgery, 25.8% of participants reported a history of hypertension, 26.7% had diabetes, and 12.2% had hypercholesterolemia. Notably, 34.4% of participants reported at least one preoperative medical condition, while 9.8% indicated other unspecified conditions (Table 5).

### 3.5. Use of Gallstone Prevention Medication

Only 40/337 (11.9%) of participants reported using medications to prevent or dissolve gallstones during the first six months following bariatric surgery, while the majority (88.1%) did not utilize prophylactic pharmacotherapy.

### 3.6. Changes in BMI Categories Pre- and Post-Bariatric Surgery

**Figure 2:**
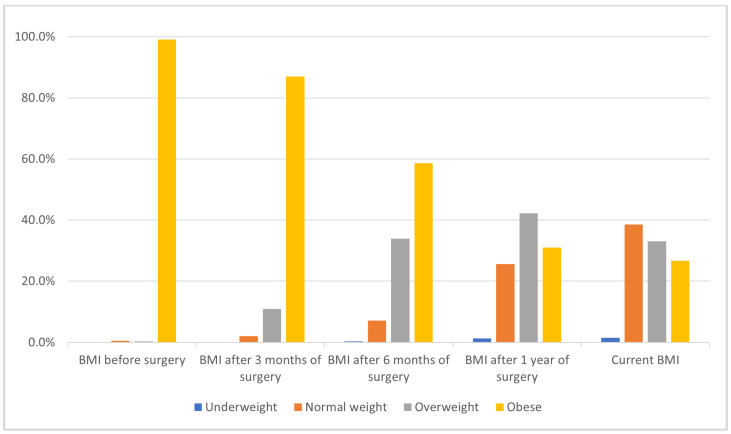
Body mass index (BMI) categories pre-/post-obesity surgery at different intervals.

The distribution of BMI categories among participants changed markedly over time following bariatric surgery, as illustrated in Figure 2 and Supplementary Table S2. Prior to surgery, nearly all patients (99.1%) were classified as obese, with only a small minority falling into the overweight (0.3%). At three months post-surgery, the proportion of obese individuals decreased to 87.0%, while the percentages of overweight (10.9%) and normal weight (2.1%) participants increased modestly. By six months, the trend toward lower BMI categories became more pronounced: 58.6% remained obese, 33.9% were overweight, 7.2% had achieved normal weight, and 0.3% were underweight. One year after surgery, nearly one-third of participants (31.0%) were still classified as obese. The majority had shifted to the overweight (42.2%) and normal weight (25.6%) categories, with a small proportion (1.3%) underweight. In the most recent assessment, only 26.7% of participants remained obese, while 33.1% were overweight, 38.6% had achieved a normal weight, and 1.5% were underweight.

### 3.7. Association of Demographic Factors with Gallstone Formation After Bariatric Surgery

Table 6 summarizes the relationship between demographic variables and the development of postoperative gallstones. Female sex was significantly associated with a higher risk of gallstone formation, with 41.5% of females developing gallstones compared to 28.8% of those without (*p* = 0.018). Age distribution also differed significantly between groups (*p* < 0.001), with patients aged 26–35 years comprising nearly half (49.8%) of those with gallstones, compared to only 23.5% in the non-gallstone group. Marital status was associated with gallstone risk (*p* = 0.006), as married individuals were more likely to develop gallstones (67.8% vs. 49.2%), while single patients were less likely (25.9% vs. 42.4%). Occupation also showed a significant association (*p* = 0.001), as employees accounted for 61.0% of gallstone cases compared to 47.7% in the non-gallstone group.

### 3.8. Association of Timing and Type of Bariatric Surgery with Gallstone Formation

Analysis showed no statistically significant association between the type of bariatric surgery performed and the subsequent development of gallstones, as the distributions of gastric sleeve, Roux-en-Y gastric bypass, adjustable gastric banding, and other procedures were similar among those with and without postoperative gallstones (*p* = 0.485) (Table 7). In contrast, the timing since surgery was significantly related to gallstone formation (*p* < 0.001). A higher proportion of patients who developed gallstones underwent surgery 7–12 months prior, with 22.4% having surgery 7–9 months earlier and 42.4% between 10 and 12 months, compared to lower rates in these intervals among those without gallstones (Table 7). These findings suggest that the risk of gallstone formation peaks between 7 and 12 months following bariatric surgery, regardless of the surgical technique used.

### 3.9. Association of Lifestyle Factors with Gallstone Formation

No significant association was observed between smoking status and gallstone formation after bariatric surgery (*p* = 0.422). However, the use of gallstone prevention medication showed a borderline significant relationship with gallstone diagnosis (*p* = 0.051) (Table 8).

### 3.10. Association of Postoperative BMI Patterns with Gallstone Development

Upon analyzing the association between the rate of body weight loss postoperatively and gallstone biogenesis, the findings suggest that rapid and substantial weight loss, particularly in participants who reached the overweight category within one year, was associated with a higher incidence of gallstone formation (50.5% vs. 28.2%). However, among those who achieved normal weight, the incidence of gallstones was not elevated (25.0% vs. 26.5%) (Table 9). BMI categories during the early postoperative period (3 and 6 months) showed no significant association with gallstone development (*p* = 0.807 and *p* = 0.114, respectively). However, BMI at one year post-surgery and current BMI demonstrated significant associations (both *p* < 0.001).

Continuous BMI analysis confirmed the above trends. The preoperative BMI showed no significant difference, but postoperative patterns diverged significantly. The group with gallstones exhibited lower BMI at 3 months, 6 months, 1 year, and currently (all *p* < 0.001) (Table 10).

### 3.11. Logistic Regression Analysis of Gallstone Risk Factors

Multivariable logistic regression identified female sex (OR: 2.62, 95% CI: 1.38–4.98, *p* = 0.003) and non-use of gallstone prevention medication (OR: 4.12, 95% CI: 1.34–12.64, *p* = 0.013) as independent predictors of gallstone formation after bariatric surgery. In contrast, the longer time since surgery (OR: 0.757, 95% CI: 0.63–0.91, *p* = 0.004) and lower current BMI (OR: 0.48, 95% CI: 0.28–0.83, *p* = 0.008) were associated with reduced risk. Other factors, including age, nationality, comorbidities, and changes in postoperative BMI, were not significantly associated with gallstone risk in the adjusted model (Table 11).

## 4. Discussion

This cross-sectional study provides important insights into gallstone incidence and associated risk factors after bariatric surgery in Northern Saudi Arabia, a region characterized by high obesity rates and a lack of localized data [21]. The findings indicate a gallstone incidence of 60.8% within the cohort, significantly exceeding the global range of 18.8–52.8% and similar to the 61.4% reported in Saudi Arabia’s Southern region [3,19,22,23,24,25]. This disparity between the identified local incidence and the global range may indicate variations in surgical practices, genetic factors, or postoperative care protocols across regions [26]. In this study, 82.4% of gallstone diagnoses were confirmed via ultrasound, consistent with international standards for gallstone detection [27]. However, the elevated incidence highlights the necessity for proactive surveillance in this population.

### 4.1. Timing and Mechanisms of Gallstone Formation

In alignment with global trends, nearly 70% of gallstones developed within the first postoperative year, peaking at 7–12 months [10,28]. This is consistent with pathophysiological models that associate rapid weight loss, evidenced by significant reductions in BMI at 3, 6, and 12 months, with cholesterol supersaturation, transient biliary stasis, and impaired gallbladder motility during the early postoperative period [29].

### 4.2. Demographics and Clinical Risk Factors

Univariate analysis revealed that female sex, age group 26–35 years, marital status (married), and occupation (employee) were significantly associated with increased gallstone risk. However, in the multivariable model, female sex remained an independent predictor (OR: 2.62, *p* = 0.003), in line with the literature attributing this to estrogen-mediated effects on cholesterol metabolism [30]. This contrasts, however, with some studies in which gender differences were less pronounced, suggesting regional variations in metabolic or cultural influences [23,31].

### 4.3. Role of Surgical Technique

Contrary to some earlier studies [32,33], the type of bariatric surgery (sleeve gastrectomy, Roux-en-Y gastric bypass, adjustable gastric banding, or other) was not significantly associated with gallstone formation (*p* = 0.811 in the adjusted model). However, the small sample size of non-sleeve procedures (e.g., Roux-en-Y: 4.5%, adjustable banding: 3.6%) limits the statistical power to detect procedural differences. While this finding suggests that patient-specific or metabolic factors may outweigh surgical technique in this population, further studies with larger, balanced cohorts are needed to confirm these observations.

### 4.4. BMI Patterns and Weight Loss

Rapid and substantial postoperative weight loss, as reflected by lower BMI at one year and the most recent follow-up, was associated with gallstone formation in univariate analysis. However, a more detailed examination of BMI categories at one year revealed that the risk of gallstones was highest among patients who transitioned from obesity to the overweight category, while those who achieved normal weight did not have an increased risk compared to those who remained obese. This pattern suggests that the magnitude and rapidity of BMI reduction (i.e., BMI excursion), rather than simply the final BMI category, may play an important role in gallstone pathogenesis. Patients who became overweight may have experienced more abrupt or substantial weight loss, increasing their susceptibility to gallstone formation, whereas those who achieved normal weight might have lost weight more gradually or maintained a healthier metabolic adaptation. In the adjusted model, lower current BMI and longer time since surgery were associated with reduced risk of gallstones. This apparent paradox may be explained by the initial lithogenic effect of rapid weight loss, followed by risk attenuation as weight stabilizes and metabolic adaptation occurs over time. Thus, the period of greatest risk is during the rapid weight loss phase, while sustained lower BMI is ultimately protective. This observation supports the hypothesis that not only the endpoint of weight loss but also the trajectory and rate of BMI change are critical determinants of gallstone risk after bariatric surgery.

### 4.5. Prophylactic Challenges

Despite guidelines recommending UDCA prophylaxis and promising results associated with its use for gallstone resolution after bariatric surgery [34,35,36,37], only 11.9% of patients reported using gallstone prevention medication postoperatively. This mirrors other studies where UDCA adherence was suboptimal [28,38]. Non-use of prophylaxis was a strong independent risk factor for gallstone formation (OR: 4.12, *p* = 0.013), emphasizing the need for greater adherence to preventive strategies. Meta-analysis of randomized control studies demonstrates UDCA reduces cholecystectomy rates and gallstone incidence at three, six, and twelve months when administered prophylactically, emphasizing the need for standardized protocols in Saudi bariatric centers [39]. Additionally, probiotics (e.g., Lactobacillus) and dietary interventions (e.g., omega-3 supplementation) show promise in modulating bile composition and gut microbiota, offering safer alternatives for long-term management [40,41,42,43].

### 4.6. Other Factors

Comorbidities (e.g., hypertension, diabetes), smoking status, and nationality were not significantly associated with gallstone risk in this cohort, highlighting the predominance of sex, weight loss dynamics, and prophylaxis use as the main determinants.

### 4.7. Clinical and Policy Implications

Given the high incidence and early onset of gallstones, routine ultrasound screening during the first postoperative year is warranted, especially for high-risk groups (females, those with rapid weight loss, and non-users of prophylaxis). Regional guidelines should prioritize early identification and targeted prevention, including patient education and improved access to UDCA and addressing barriers like cost and side effects through subsidized programs or combination therapies with dietary modifications.

### 4.8. Limitations and Future Directions

This study has several limitations. First, its cross-sectional design precludes causal inferences. Second, the use of convenience sampling and self-reported weights may introduce selection bias (e.g., underrepresentation of certain subgroups) and measurement bias, respectively. Third, the study population, drawn from a single region in Northern Saudi Arabia, may not be fully representative of the broader Saudi or global population, limiting generalizability.

### 4.9. Future Directions

Future research should adopt prospective designs to (1) track long-term outcomes, such as the progression of asymptomatic gallstones to cholecystitis or pancreatitis, (2) compare UDCA dosing regimens (500 mg vs. 250 mg) in Arab cohorts to optimize adherence and efficacy [35], and (3) investigate gut microbiota dynamics (e.g., Ruminococcus and Lactobacillus ratios) as predictive biomarkers for gallstone formation [3].

## 5. Conclusions

This study highlights a critical public health gap in Northern Saudi Arabia, where the incidence of gallstones after bariatric surgery exceeds global averages. The principal independent risk factors identified were female sex, rapid postoperative weight loss, and lack of prophylactic medication use. While no statistically significant association was observed between the type of bariatric surgery and gallstone risk in this cohort, this finding should be interpreted with caution due to the small number of patients undergoing procedures other than sleeve gastrectomy. Larger, more balanced studies are needed to clarify the impact of surgical techniques. These findings underscore the need for routine postoperative surveillance, targeted patient education, and improved adherence to gallstone prevention strategies, particularly the use of ursodeoxycholic acid, during the high-risk period after surgery. Implementing region-specific guidelines and accessible prophylactic measures can help reduce gallstone-related morbidity and improve long-term outcomes for bariatric patients in this high-risk region.

## Figures and Tables

**Table 1:** The characteristics of the enrolled study participants, including those who underwent weight loss surgery.

Variables		Total Recruited Study Participants (*n* = 509)		Participants Who Underwent Obesity Surgery (*n* = 337)	
		Frequency	Percent (%)	Frequency	Percent (%)
Sex	Male	314	61.7	214	63.5
	Female	195	38.3	123	36.5
Age	19–25	139	27.3	85	25.2
	26–35	182	35.8	133	39.5
	36–45	111	21.8	78	23.1
	46–60	77	15.1	41	12.2
Nationality	Saudi	481	94.5	319	94.7
	Non-Saudi	28	5.5	18	5.3
Educational Level	Uneducated	15	2.9	8	2.4
	Primary	10	2.0	5	1.5
	Intermediate	6	1.2	3	0.9
	Secondary	64	12.6	43	12.8
	Diploma	104	20.4	75	22.3
	Bachelor’s	279	54.8	182	54.0
	Postgraduate	31	6.1	21	6.2
Marital Status	Single	176	34.6	109	32.3
	Married	300	58.9	204	60.5
	Divorced	27	5.3	21	6.2
	Widowed	6	1.2	3	0.9
Occupation	Student	115	22.6	74	22.0
	Employee	282	55.4	188	55.8
	Unemployed	70	13.8	53	15.7
	Retired	34	6.7	17	5.0
	Other	8	1.6	5	1.5

Data are presented as frequencies and percentages (%).

**Table 2:** Frequencies of timing and type of obesity surgery.

Timing and Type of Obesity Surgery (*n* = 337)		Frequency	Percent (%)
When did you have obesity surgery?	1–3 Months	21	6.2
	4–6 Months	15	4.5
	7–9 Months	68	20.2
	10–12 Months	110	32.6
	13–18 Months	42	12.5
	19–24 months	26	7.7
	More than 24 Months	55	16.3
What type of surgery did you have?	Gastric Sleeve Surgery	298	88.4
	Roux-en-Y Gastric Bypass Surgery (Roux-en-Y)	15	4.5
	Adjustable Gastric Banding Surgery	12	3.6
	Others †	12	3.6

Data are presented as frequencies and percentages (%). † “Others” include less commonly performed surgical procedures in the study area: fundoplication (n = 4), mini-gastric bypass (n = 3), vertical banded gastroplasty (n = 3), and gastric plication (n = 2), each with less than five cases. Grouped to mitigate small-sample bias.

**Table 3:** Frequencies of gallbladder conditions in pre-/post-obesity surgery.

Pre- and Post-Obesity Surgery Gallbladder Conditions (*n* = 337)	No *n* (%)	Yes *n* (%)
Have you been diagnosed with gallstones or had your gallbladder removed before obesity surgery?	337 (100.0%)	0 (0.0%)
Did you have an ultrasound (sonar) before obesity surgery?	145 (43.0%)	192 (57.0%)
Have you been diagnosed with gallstones after undergoing obesity surgery?	132 (39.2%)	205 (60.8%)
If the answer to the previous question is yes, were you diagnosed with gallstones through ultrasound (sonar)?	Frequency	Percent (%)
No	36	17.6
Yes	169	82.4
Total	205	100.0

Data are presented as numbers (*n*)/frequencies and percentages (%).

**Table 4:** Timing of gallstone development after obesity surgery.

When Did You Develop Gallstones After Surgery?	Frequency	Percent (%)	*p*-Value
1–3 months	18	8.8	<0.001 *
4–6 months	42	20.5	
7–9 months	93	45.4	
9–12 months	30	14.6	
1.5 years	6	2.9	
2 years	6	2.9	
More than 2 years	10	4.9	
Total	205	100.0	

Data are presented as frequencies and percentages (%). The chi-square test was employed. * The significance was set at *p*-values ≤ 0.05.

**Table 5:** Pre-obesity surgery medical conditions.

Did You Have Any of the Following Diseases Before the Operation?	No *n* (%)	Yes *n* (%)
High blood pressure	250 (74.2%)	87 (25.8%)
Diabetes	247 (73.3%)	90 (26.7%)
High cholesterol	296 (87.8%)	41 (12.2%)
Anemia	329 (97.6%)	8 (2.4%)
Thyroid diseases	318 (94.4%)	19 (5.6%)
Asthma or chronic lung disease	327 (97.0%)	10 (3.0%)
Chronic heart diseases	328 (97.3%)	9 (2.7%)
Chronic liver diseases	330 (97.9%)	7 (2.1%)
Chronic kidney diseases	328 (97.3%)	9 (2.7%)
No, I don’t have any of them	221 (65.6%)	116 (34.4%)
Other	304 (90.2%)	33 (9.8%)

Data are presented as numbers (*n*)/frequencies and percentages (%).

**Table 6:** Association of demographic factors with gallstone formation after obesity surgery.

		Diagnosis of Gallstones After Undergoing Obesity Surgery				*χ* ^2^	*p*-Value
		No (*n* = 132)		Yes (*n* = 205)			
		n	%	n	%		
Sex	Male	94	71.2%	120	58.5%	5.566	0.018 *
	Female	38	28.8%	85	41.5%		
Age	19–25	49	37.1%	36	17.6%	27.820	<0.001 *
	26–35	31	23.5%	102	49.8%		
	36–45	36	27.3%	42	20.5%		
	46–60	16	12.1%	25	12.2%		
Nationality	Saudi	126	95.5%	193	94.1%	0.272	0.602
	Non-Saudi	6	4.5%	12	5.9%		
Educational Level	Uneducated	1	0.8%	7	3.4%	6.774	0.342
	Primary	1	0.8%	4	2.0%		
	Intermediate	1	0.8%	2	1.0%		
	Secondary	19	14.4%	24	11.7%		
	Diploma	30	22.7%	45	22.0%		
	Bachelor’s	68	51.5%	114	55.6%		
	Postgraduate	12	9.1%	9	4.4%		
Marital Status	Single	56	42.4%	53	25.9%	12.459	0.006 *
	Married	65	49.2%	139	67.8%		
	Divorced	9	6.8%	12	5.9%		
	Widowed	2	1.5%	1	0.5%		
Occupation	Student	40	30.3%	34	16.6%	18.321	0.001 *
	Employee	63	47.7%	125	61.0%		
	Unemployed	17	12.9%	36	17.6%		
	Retired	7	5.3%	10	4.9%		
	Other	5	3.8%	0	0.0%		

Data are presented as frequencies and percentages (%). The chi-square test or Fisher’s exact test was employed. * The significance was set at *p*-values ≤ 0.05.

**Table 7:** Association of the type/time of obesity surgery with gallstone formation after surgery.

Variables		Diagnosis of Gallstones After Undergoing Obesity Surgery				*χ* ^2^	*p*-Value
		No (*n* = 132)		Yes (*n* = 205)			
		*n*	%	*n*	%		
Type of surgery	Gastric Sleeve Surgery	119	90.2%	179	87.3%	2.449	0.485
	Roux-en-Y Gastric Bypass Surgery (Roux-en-Y)	7	5.3%	8	3.9%		
	Adjustable Gastric Banding Surgery	3	2.3%	9	4.4%		
	Other	3	2.3%	9	4.4%		
When did you have obesity surgery?	1–3 Months	16	12.1%	5	2.4%	48.264	<0.001 *
	4–6 Months	10	7.6%	5	2.4%		
	7–9 Months	22	16.7%	46	22.4%		
	10–12 Months	23	17.4%	87	42.4%		
	13–18 Months	14	10.6%	28	13.7%		
	19–24 months	18	13.6%	8	3.9%		
	More than 24 Months	29	22.0%	26	12.7%		

Data are presented as frequencies and percentages (%). The chi-square test or Fisher’s exact test was employed. * The significance was set at *p*-values ≤ 0.05.

**Table 8:** Association of smoking habits and gallstone prevention medication use with gallstone formation after obesity surgery.

Variables		Diagnosis of Gallstones After Undergoing Obesity Surgery				*χ* ^2^	*p*-Value
		No (*n* = 132)		Yes (*n* = 205)			
		n	%	n	%		
Are you a smoker?	No	85	64.4%	137	66.8%	1.036	0.596
	Yes	41	31.1%	55	26.8%		
	Former smoker	6	4.5%	13	6.3%		
Gallstone Prevention Medication Use	No	122	92.4%	175	85.4%	3.824	0.051 *
	Yes	10	7.6%	30	14.6%		

Data are presented as frequencies and percentages (%). The chi-square test was employed. * The significance was set at *p*-values ≤ 0.05.

**Table 9:** Association of BMI at different intervals with gallstone formation after obesity surgery.

Variables		Diagnosis of Gallstones After Undergoing Obesity Surgery				*χ* ^2^	*p*-Value
		No (*n* = 132)		Yes (*n* = 205)			
		n	%	n	%		
BMI before surgery	Underweight	0	0.0%	0	0.0%	0.743	0.690
	Normal weight	1	0.8%	1	0.5%		
	Overweight	0	0.0%	1	0.5%		
	Obese	131	99.2%	203	99.0%		
BMI after the first three months of surgery	Underweight	0	0.0%	0	0.0%	0.429	0.807
	Normal weight	2	1.6%	5	2.5%		
	Overweight	15	11.6%	21	10.4%		
	Obese	112	86.8%	176	87.1%		
BMI after six months of surgery	Underweight	0	0.0%	1	0.5%	5.959	0.114
	Normal weight	12	9.2%	12	5.9%		
	Overweight	35	26.9%	78	38.4%		
	Obese	83	63.8%	112	55.2%		
BMI after one year of surgery	Underweight	0	0.0%	4	2.0%	23.439	<0.001 *
	Normal weight	31	26.5%	49	25.0%		
	Overweight	33	28.2%	99	50.5%		
	Obese	53	45.3%	44	22.4%		
Current BMI	Underweight	1	0.8%	4	2.0%	21.278	<0.001 *
	Normal weight	38	29.7%	89	44.3%		
	Overweight	37	28.9%	72	35.8%		
	Obese	52	40.6%	36	17.9%		

Data are presented as frequencies and percentages (%). The chi-square test was employed. * The significance was set at *p*-values ≤ 0.05. BMI: Body mass index.

**Table 10:** Comparison of BMI at different intervals and gallstone formation after obesity surgery.

BMI at Different Intervals	Diagnosis of Gallstones After Undergoing Obesity Surgery		*p*-Value
	No	Yes	
BMI before surgery	44.2 ± 6.8	45.0 ± 8.1	0.332
BMI after the first three months of surgery	37.0 ± 6.6	35.1 ± 5.5	* 0.005
BMI after six months of surgery	33.0 ± 6.6	30.9 ± 4.8	<0.001 *
BMI after one year of surgery	30.3 ± 7.0	27.3 ± 4.1	<0.001 *
Current BMI	29.1 ± 6.9	26.1 ± 4.3	<0.001 *

Data are presented as mean ± standard deviation (SD). Student *t*-test was employed. * The significance was set at *p*-values ≤ 0.05.

**Table 11:** Logistic regression analysis of factors associated with gallstone formation after obesity surgery.

	Wald	*p-*Value	Odds Ratio	95% Confidence Interval	
				Lower	Upper
Sex	8.735	0.003 *	2.62	1.384	4.977
Age	1.899	0.168	1.32	0.888	1.971
Nationality	0.041	0.840	0.85	0.184	3.964
Educational level	2.789	0.095	0.79	0.606	1.041
Marital status	2.912	0.088	1.71	0.923	3.172
Occupation	2.983	0.084	0.69	0.455	1.051
When did you have obesity surgery	8.494	0.004 *	0.76	0.628	0.913
Type of surgery	0.057	0.811	1.06	0.656	1.715
High blood pressure	0.012	0.914	1.05	0.455	2.410
Diabetes	0.737	0.391	0.69	0.292	1.619
High cholesterol	0.619	0.432	1.56	0.516	4.713
Anemia	0.891	0.345	0.37	0.048	2.888
Thyroid diseases	0.003	0.955	0.96	0.223	4.122
Asthma or chronic lung disease	0.782	0.376	2.10	0.263	34.072
Chronic heart diseases	0.488	0.485	2.14	0.253	18.088
Chronic liver diseases	0.000	0.985	0.98	0.093	10.294
Chronic kidney diseases	0.007	0.933	0.93	0.188	4.632
No, I don’t have any of them	3.092	0.079	0.41	0.150	1.108
Other	0.519	0.471	0.66	0.209	2.061
Smoking Habits	0.917	0.338	1.28	0.775	2.099
Gallstone Prevention Medication Use	6.108	0.013 *	4.12	1.340	12.643
BMI before surgery	0.638	0.424	1.90	0.393	9.220
BMI after the first three months of surgery	0.505	0.478	1.39	0.559	3.470
BMI after six months of surgery	0.002	0.963	0.98	0.480	2.014
BMI after one year of surgery	0.179	0.672	0.87	0.456	1.659
Current BMI	6.992	0.008 *	0.48	0.276	0.826

* The significance was set at *p*-values ≤ 0.05.

## Data Availability

The original contributions presented in this study are included in the article. Further inquiries can be directed to the corresponding authors.

## Supplementary Materials

The following supporting information can be downloaded at https://www.mdpi.com/article/10.3390/clinpract15070115/s1. Table S1: Descriptive statistics for height and weight and BMI for the study participants; Table S2: Body mass index (BMI) categories pre-/post-obesity surgery.

## Abbreviations

The following abbreviations are used in this manuscript:

BMI	body mass index
RYGB	Roux-en-Y Gastric Bypass Surgery
SG	sleeve gastrectomy
STROBE	STrengthening the Reporting of OBservational studies in Epidemiology
UDCA	ursodeoxycholic acid
