# Supplementary material for: Incidence of Gallstones in Patients with Obesity After Bariatric Surgery in Northern Saudi Arabia: A Cross-Sectional Study
Source: Clin Pract. 2025 Jun 23;15(7):115. doi: 10.3390/clinpract15070115 (PMC12293454; doi:10.3390/clinpract15070115)
Supplement: Supplementary file 1 [file clinpract-15-00115-s001.zip › clinpract-3664016-supplementary.pdf]

Incidence of Gallstones in Patients with Obesity after Bariatric Surgery in Northern Saudi Arabia: A Cross-Sectional Study"

Table S1. Descriptive Statistics for height and weight and BMI for the study participants.

	N	Minimum	Maximum	Mean	Std. Deviation
Height (in centimeters)	337	148	193	170.02	9.260
**Weight (in kilograms) at different intervals**					
Weight before surgery	337	77	222	129.93	24.199
Weight after the first three months of surgery	336	53	176	103.61	19.814
Weight after six months of surgery	333	49	150	91.84	18.498
Weight after one year of surgery	316	50	148	82.26	17.574
Current weight	335	50	148	78.65	17.391
**BMI at different intervals**					
BMI before surgery	337	21.7	86.3	44.7	7.6
BMI after the first three months of surgery	336	18.7	55.4	35.8	6.0
BMI after six months of surgery	333	17.6	53.1	31.7	5.7
BMI after one year of surgery	316	14.8	53.1	28.4	5.6
Current BMI	335	14.8	53.1	27.2	5.6

N: number; Std.: Standard deviation.

Table S2. Body mass index (BMI) categories pre-/post-obesity surgery

	**Underweight**	**Normal weight**	**Overweight**	**Obese**	**Total**
BMI before surgery	0 (0.0%)	2 (0.6%)	1 (0.3%)	334 (99.1%)	337
BMI after the first three months of surgery	0 (0.0%)	7 (2.1%)	36 (10.9%)	288 (87.0%)	331
BMI after six months of surgery	1 (0.3%)	24 (7.2%)	113 (33.9%)	195 (58.6%)	333
BMI after one year of surgery	4 (1.3%)	80 (25.6%)	132 (42.2%)	97 (31.0%)	313
Current BMI	5 (1.5%)	127 (38.6%)	109 (33.1%)	88 (26.7%)	329
